# Preparation and characterization of Zein‐sulfated *Cardamine hupingshanensis* polysaccharide composite films

**DOI:** 10.1002/fsn3.2625

**Published:** 2021-10-29

**Authors:** Zimu Zhang, XiuFang Huang, ShiChan Li, Chi Zhang, Kai Luo

**Affiliations:** ^1^ College of Biology and Science Technology Hubei min Zu University Enshi China

**Keywords:** antioxidant, composite film, sulfated *Cardamine hupingshanensis* polysaccharide

## Abstract

*Cardamine hupingshanensis* polysaccharide (CHP) was modified by the sulfur trioxide–pyridine method to obtain the sulfated *C*. *hupingshanensis* polysaccharide (SCHP) with a substitution degree of 0.72. The spectral results revealed that the sulfate group was successfully introduced to CHP. In the in vitro antioxidant assay, SCHP showed the highest scavenging rate of hydroxyl radicals, ABTS, and DPPH. Different concentrations of SCHP were chosen to form a compound with Zein to prepare novel bioactive films successfully. The functional and characterization studies of the films were also conducted. The scavenging ability of the films for hydroxyl radicals, ABTS, and DPPH was improved by adding different concentrations of SCHP. Although the films showed a decrease in transparency with the addition of 4 mg/ml SCHP, there was an improvement in tensile strength compared to films without the addition of SCHP. These findings indicate that Zein‐SCHP films can be used as a functional food packaging material with antioxidant properties.

## Graphical abstract

Edible composite films were produced using sulfated *Cardamine hupingshanensis* polysaccharides. The composite film has good antioxidant function.

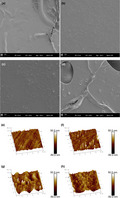

## 1 INTRODUCTION

Currently synthetic plastic food packaging materials have an increasingly negative impact on the environment. There is a significant demand for biodegradable films made of natural polymers with additional environmental benefits to decrease the burden of plastic packaging on the environment. Common biofilm materials under investigation include carbohydrates, proteins, and lipids (Falguera et al., 2011). Protein‐based membranes are usually superior to polysaccharide‐based membranes because of their structural specificity and ability to form stronger intermolecular covalent bonds (Cuq et al., 1995). These proteins possess outstanding film‐building capabilities, high optical clarity, high elasticity, and gas barrier properties (Chiou et al., 2008). Zein, the major storage protein in maize endosperm, is a hydrophobic protein (Kriz & Larkins, 2008) that has been actively investigated for its membrane‐forming ability. Zein films show outstanding water resistance and have been used to produce hydrophobic films (Gu et al., 2013), which makes it possible to incorporate it into biodegradable membranes as a hydrophobic component to improve the water resistance of the membrane. However, single maize alcoholic protein films with a single function do not have strong bioactive functions. Plant polysaccharides have been extensively researched in food and biomedical fields for their various functions and biological activities, such as antioxidant, anticoagulant, hypoglycemic, immunomodulatory, and antitumor (Li et al., 2019). The chemical modification of polysaccharides can improve their natural biological effects and sometimes lead to novel functional properties (Deng et al., 2015). Li et al., (2020) successfully attached sulfate groups to the long chains of *Russula virescens* polysaccharides and obtained five sulfated *R*. *virescens* polysaccharides with different degrees of substitution. The sulfated *R*. *virescens* polysaccharides showed good antioxidant, anticoagulant, antibacterial, and antitumor activities in in vitro activity tests. Sulfation of polysaccharides enhances their water solubility and modifies the conformation of polysaccharide chains; thus, changing their biological activity. Therefore, chemically modified polysaccharides were used to cross‐link with Zein to make an edible food packaging material with certain functionality.

*Cardamine hupingshanensis* is a species in the family Brassicaceae of crushed rice capers. It is mainly distributed in the Hunan Province and Hubei Province of China. It is known mainly for its selenium‐rich properties. Studies on *C*. *hupingshanensis* are currently focused on the antioxidant activity of flavonoids and the extraction method of polysaccharides. Li et al. (2021) extracted a single component of *C*. *hupingshanensis* polysaccharide (CHP) using column chromatography and the monosaccharide composition of CHP was determined using NMR and HPLC. It was observed that CHP had good therapeutic effects on diabetic mice. The results demonstrated that CHP had hypoglycemic and antioxidant effects. Currently, no sulfation modification of CHP has been reported in the literature. Therefore, in this study, sulfated *C*. *hupingshanensis* polysaccharide (SCHP) was added to Zein to produce an edible composite membrane with antioxidant properties. Moreover, the changes in mechanical properties, structural properties, and biological activities of SCHP‐containing membranes were investigated.

## 2 MATERIALS AND METHODS

*Cardamine hupingshanensis* was purchased from Shuanghe Township in Enshi (Hu Bei, China). SO3·Pyr complex and DMSO were purchased from Sigma‐Aldrich. Diethylaminoethyl cellulose (DEAE‐52) and food grade Zein were procured from Shanghai Yuanye Technology Limited. All the other chemicals were of analytical grade and no further refining was required.

### 2.1 Preparation of crude *C. hupingshanensis*

The purchased *C*. *hupingshanensis* was first dried and then powdered using an ultramicropulverizer. The powder was then mixed thoroughly with distilled water (1:25) and then cooked at 75°C for 4 h. The filtrate was retained by straining (200‐mesh sieve). The filtrate was concentrated under reduced pressure to 20% of its original volume, and after several rounds of protein removal, anhydrous ethanol was added for alcohol precipitation. The crude CHP was obtained by centrifugation after standing overnight at 4°C. The crude CHP was then dissolved in distilled water (0.4 g/ml) and injected into a DEAE‐52 column (3.5 × 40 cm, Anhui Weiss Experimental Equipment Co.). The column was subsequently eluted with distilled water. The filtrate was then collected, concentrated, and lyophilized to obtain CHP.

### 2.2 Sulfation of CHP

About 0.4 g of CHP was mixed with 100 ml of DMSO for 1 h at 20°C, after which it was sulfated using the procedure described by Liu, Qin, et al. (2019) and left for the complete dispersion of CHP in DMSO. Then, SO3·Pyr complex (CHP: SO3‐Pry = 0.2:5 g/g) was added, and the mixture was stirred at 55°C for 2 h. When the reaction is complete, 1 M NaOH solution is added dropwise in an ice water bath until pH = 7. Then, the mixture was dialyzed with a 3500‐Da dialysis bag for 5 days. The dialysate was then removed by rotary evaporation, centrifuged, and finally freeze dried after overnight alcohol deposition to obtain SCHP.

### 2.3 Determination of the degree of substitution (DS) of SCHP

The sulfur content (S%) of SCHP was estimated by the barium chloride–gelatin method, and a standard curve was established using sodium sulfate as the standard. DS is the average number of sulfate groups on each SCHP, calculated from the sulfur content.

### 2.4 Preparation of Zein‐SCHP film

The principle of liquid–liquid dispersion was used to make the Zein‐SCHP protein membrane. SCHP (0 mg/ml, 0.02 mg/ml, 0.03 mg/ml, and 0.04 mg/ml) and glycerol (8%, v/v) were mixed in 2 ml of distilled water. Zein (150 mg/ml) was dissolved in 8 ml of 75% ethanol and stirred electromagnetically for 30 min. While stirring, the SCHP and glycerol mixture was slowly dripped into the Zein solution. The mixture was then sonicated for 0.5 min to remove any air bubbles. About 7 ml of the film‐forming solution was poured into a polyethylene mold of a diameter of 8 cm to control the thickness of the film. Zein films without SCHP and those containing different concentrations of SCHP (designated as Zein film, Zein‐SCHP_2_, Zein‐SCHP_3_, and Zein‐SCHP_4_, respectively) were prepared. The films were allowed to dry at 15°C for 24 h and held at 25°C and 50% relative humidity for 24 h before the final experiments.

### 2.5 Mechanical properties of Zein‐SCHP film

The film was cut into long strips of 1 cm × 6 cm before measurement and equilibrated for 24 h at 25°C and 50% humidity. The test conditions were tensile speed at 10 mm/min and an initial length of 35 mm. The tensile strength and elongation at break were calculated according to the following equations:

(1) $$\text{TS}=\frac{F}{S}$$

where *F* is the maximum tensile force at rupture (N) and *S* is the cross‐sectional area of Zein‐SCHP film.

(2) $$E=\frac{L‐L_{0}}{L_{0}}$$

where *E* is the Zein‐SCHP film elongation at break, *L* is the length of Zein‐SCHP membrane at fracture, and *L*_0_ is the initial length of Zein‐SCHP membrane.

### 2.6 Spectroscopic analysis of Zein‐SCHP films

The analyses were conducted using a Nicolet iS5 Fourier transform infrared spectrometer (FTIR) with a sweeping range of 4000–400 cm^−1^ (Thermo Scientific). The dried Zein‐SCHP films were mixed with KBr at a ratio of 1:150 w/w and dried under vacuum for 1 h. The films were then pressed into thin slices for FTIR measurements.

### 2.7 Color and opacity of Zein‐SCHP films

The color of the film was detected using a benchtop spectrophotometer. The CS‐820N benchtop spectrophotometer (China Color Spectrum Technology Co., Ltd.) was used to detect five random points on the film and calculate the average value of the data.

The Zein‐SCHP films were cut into 1 cm × 4.5 cm strips. The cut films were then equilibrated at 25°C and 50% humidity for 24 h. The equilibrated films were placed in a quartz cuvette to detect the absorbance values at 600 nm, and the opacity was calculated. The relationship between opacity and thickness is expressed as follows:

(3) $$\text{Relationship}\;l\frac{\text{Abs}}{T}$$

where *T* is the thickness of the Zein‐SCHP film.

### 2.8 Measurement of water vapor transmission rate and water contact angle

The measurements were carried out according to the method of Cao and Song (2019) with some modifications. Briefly, 10 ml of distilled water was added to a permeable cup with an inner diameter of 2 cm, and the top of the cup was covered with a layer of sample cut into a size of 2.5 cm × 2.5 cm. The change in weight of the film was monitored every 1 h at 25°C and 50% relative humidity. After 24 h of measurement, the change in weight was calculated according to the formula.

The water contact angle was measured by the DSA100 contact angle‐measuring instrument (KRUSS, Germany) using the solid droplet method. After fixing the film on a slide, a drop of distilled water (0.1 ml) was placed on the surface of the film, and images were taken. The data were obtained by analyzing the images using the software SCA 20.

### 2.9 Thermogravimetric analysis

Thermogravimetric testing was mainly used for the analysis of the thermal stability of films, and the experiments were conducted using a TGA55 thermogravimetric analyzer (TA, USA). The films were cut to a size of 1 cm × 1 cm, placed in an aluminum crucible, and the rate of temperature increase was set to 10°C/min.

### 2.10 Scanning electron microscopy (*SEM*) and atomic force microscopy (AFM)

The surface morphology of the thin films was observed by scanning electron microscopy (*SEM*). The film surface was first gold plated and observed using a ZEISS Gemini 300 scanning electron microscope (Zeiss, Germany) at an accelerating voltage of 5 kV.

An atomic force microscope was used to observe the roughness of the film surface. First, a special double‐sided adhesive was used to fix the film, and the film surface was scanned using a Bruker Dimension ICON (Bruker, Germany).

### 2.11 In vitro antioxidant assay of Zein‐SCHP films

First, 100 mg of SCHP films containing different concentrations of SCHP were dried, weighed, and dissolved in 5 ml of distilled water. After shaking well, the supernatant was separated to measure the antioxidant activity of the Zein‐SCHP films. The 2,2‐azido‐*bis*‐3‐ethylbenzothiazoline‐6‐sulfonic acid (ABTS) and 1,1‐diphenyl‐2‐picrylhydrazyl (DPPH) radicals were detected by the method of Li et al. (2019). The Zein‐SCHP membrane solution (0.2 ml) was mixed with 4 ml of ABTS solution, and the absorbance was measured at 734 nm after 300 s of reaction in a dark environment. Zein‐SCHP membrane solution (2 ml) was mixed with DPPH solution (2 ml), and the absorbance was measured at 517 nm after 30 min of reaction in a dark environment. The hydroxyl radical scavenging rate was measured according to the method of Xiong et al. (2019). Zein‐SCHP membrane solution (2 ml) was mixed with ferrous sulfate solution (2 ml), salicylic acid ethanol solution (2 ml), and hydrogen peroxide (2 ml) and absorbance values were measured at 510 nm after a water bath for 30 min (37°C).

### 2.12 Data analysis

Data from all of the above experiments were averaged after three or five independent replicates and analyzed using Origin Pro2020 software and SPSS 25 software.

## 3 RESULTS AND DISCUSSION

### 3.1 DS analysis

The standard curve was measured according to the above method and the average value was taken three times. The regression equation of sulfate and absorbance was *y* = 1.3417*x* + 0.024, *R* ^2^ was 0.9921, and the linearity of the equation was good. The degree of substitution of the sample SCHP was 0.72 according to the above equation, and the results showed that the CHP sulfation reaction was successful, in which the degree of substitution of unmodified CHP was 0.

### 3.2 Analysis of film‐forming properties

In the preliminary experiments, it was observed that SCHP did not precipitate polysaccharides after slowly mixing with Zein solution. This may be because CHPs show greatly improved water solubility after sulfation. Furthermore, due to the slow addition of SCHP solution to Zein, the maize alcoholic protein encapsulated the SCHP during the dissolution process. Similar results were observed for the dissolution of soybean polysaccharides in Zein using the liquid–liquid dispersion method by Li et al. (2019).

### 3.3 Mechanical properties of Zein‐SCHP films

The thickness of the film was observed to increase with the increase of SCHP concentration (Table 1). Also, regarding mechanical properties, the tensile strength of the films increased with an increase in SCHP addition which was mainly due to the increased intermolecular forces between SCHP and Zein during the film‐formation process. Young's modulus decreased with an increase in SCHP addition, indicating that the addition of SCHP facilitated the increase in film flexibility. This favors the application of the films in food packaging. These findings are in line with the results of Liu et al. (2019) on soybean polysaccharide films. The water vapor transmission (WVP) of the films containing SCHP was larger compared to those without SCHP. The WVP of Zein‐SCHP films increased with an increasing concentration of SCHP, which may be due to the comparatively weak film substrate formed after the addition of SCHP during the process of film formation. It led to fine pores on the film surface that provided channels for the transfer of water molecules. Similar results were observed in alginate films from *Sargassum fulvellum* extract by Kim et al. (2018).

**TABLE 1:** Physical properties of Zein‐SCHP films

Film	Thickness (mm)	Tensile strength (MPa)	Elongation at break (%)	Young's modulus (MPa)	WVP (g/m^2^·day)	Contact angle (◦)
Zein‐film	0.1252 ± 0.012	0.5307 ± 0.161	0.1844 ± 0.159	300.9 ± 9.43	54.34 ± 8.65	40.3 ± 0.1
Zein‐SCHP_2_	0.1718 ± 0.007	0.6388 ± 0.198	0.2509 ± 0.054	278.34 ± 7.96	62.52 ± 5.62	59.4 ± 0.2
Zein‐SCHP_3_	0.1732 ± 0.008	0.7201 ± 0.563	0.3143 ± 0.257	235.65 ± 6.46	90.12 ± 4.32	68.9 ± 1.2
Zein‐SCHP_4_	0.1744 ± 0.0092	0.8711 ± 0.152	0.676 ± 0.286	117.25 ± 3.16	95.66 ± 7.65	80.2 ± 0.8

Note

Mean ± *SD*, *n* = 3

The contact angle of Zein‐SCHP film water is presented in Table 1. The contact angle increased in size with an increase in the concentration of SCHP. This indicated that the addition of SCHP enhanced the hydrophobicity of the film surface. This occurrence is more favorable for application in the food industry. This may be attributed to the increased water solubility of the polysaccharides from the sulfated modified pot bottle‐crushed rice capsule and the combination of the hydrophobic layer of Zein with the hydrophilic layer of SCHP during film formation. This led to the exposure of the hydrophobic layer of Zein, leading to the enhanced hydrophobicity of the film surface. This resulted in a structure with a hydrophobic shell of Zein wrapped around a hydrophilic core of SCHP.

### 3.4 Thermogravimetric (TG) analysis of Zein‐SCHP films

**FIGURE 1:**
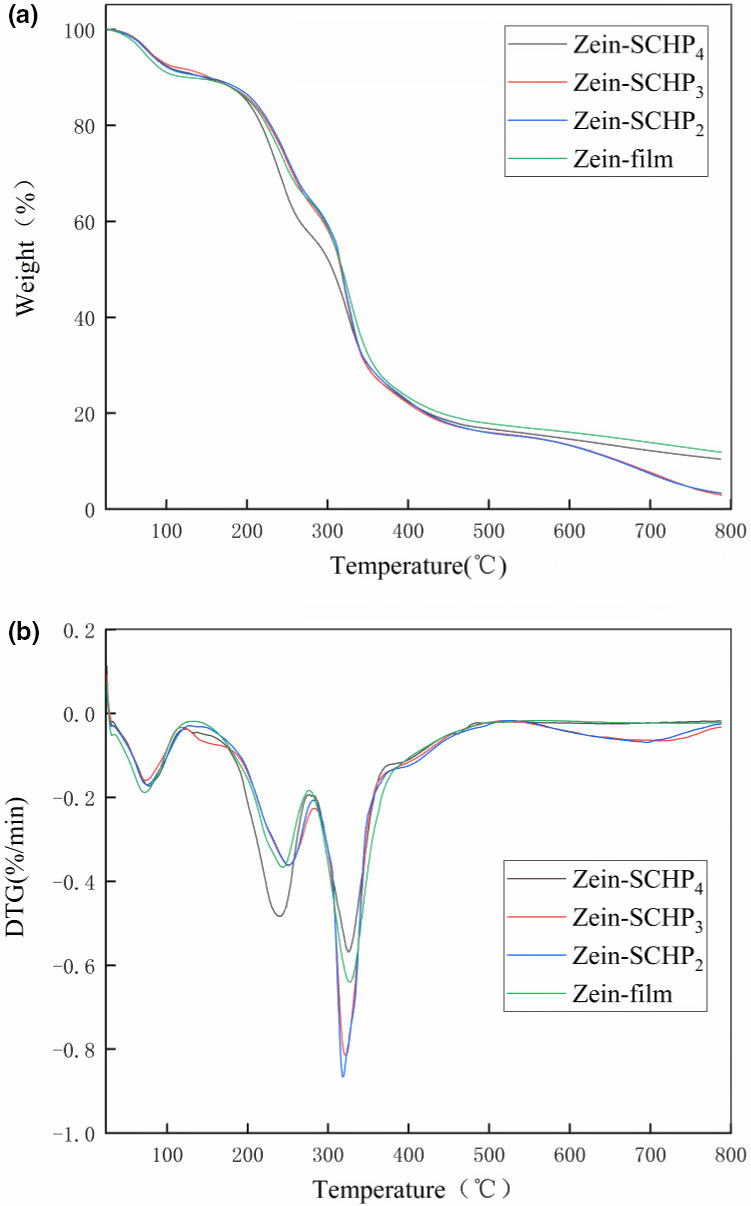
TGA (a) and DTG (b) curves of Zein‐SCHP films

The thermogravimetric analysis curve indicated that the loss of film mass under heat was mainly divided into three stages (Figure 1). The temperature during the first stage of loss was 30–145°C, and the reason for this loss was mainly due to the evaporation of water and ethanol in the film. The DTG plot indicated that the peak of the first stage increased from 72°C to 79°C, which mainly indicated that the increase of SCHP led to a closer bonding between the polar molecules and water molecules in the film. This is in line with the results of Liu, Qin, et al. (2019). The second stage of loss was from 146°C to 260°C and the loss was mainly due to the decomposition of glycerol added to the film and the thermal decomposition of polysaccharides and maize alcoholic proteins. This agrees with the results of Breda et al. (2017). The second peak in DTG increased from 244°C to 251°C, indicating that the addition of SCHP increased the thermal stability of the films. However, the second peaks of Zein‐SCHP_2_, Zein‐SCHP_3_, and Zein‐SCHP4 were at 251°C, 250°C, and 248°C, respectively, which may be caused by the excess SCHP that could instead reduce the thermal stability of the films. During the third stage of loss, the temperature was 261–376°C, and the loss was mainly due to further oxidation of polysaccharides and maize alcoholic proteins in the films.

### 3.5 Optical properties of Zein‐SCHP films

The membranes containing different concentrations of SCHP and Zein films showed less variability in L* values and the values of a* and b* increased with an increasing concentration of supplemented SCHP (Table 2). This is mainly due to the dark red color of SCHP and the light‐yellow powder of Zein, as the light‐yellow color of the film solution deepens with an increase in SCHP concentration during film formation. At the same time, the transparency of the Zein film gradually decreased due to the color of SCHP.

**TABLE 2:** Optical properties of Zein‐SCHP films

Film	L*	a*	b*	Opacity (Abs/mm)
Zein‐film	35.68 ± 0.62	0.358 ± 0.15	14.82 ± 0.41	4.28 ± 0.06
Zein‐SCHP_2_	35.78 ± 0.19	1.10 ± 0.23	15.09 ± 0.40	4.84 ± 0.04
Zein‐SCHP_3_	35.97 ± 0.47	1.49 ± 0.26	16.51 ± 0.41	5.14 ± 0.5
Zein‐SCHP_4_	35.71 ± 0.49	1.98 ± 0.33	17.19 ± 0.74	5.55 ± 0.3

Note

Mean ± *SD*, *n* = 5

### 3.6 FTIR analysis of Zein‐SCHP films

**FIGURE 2:**
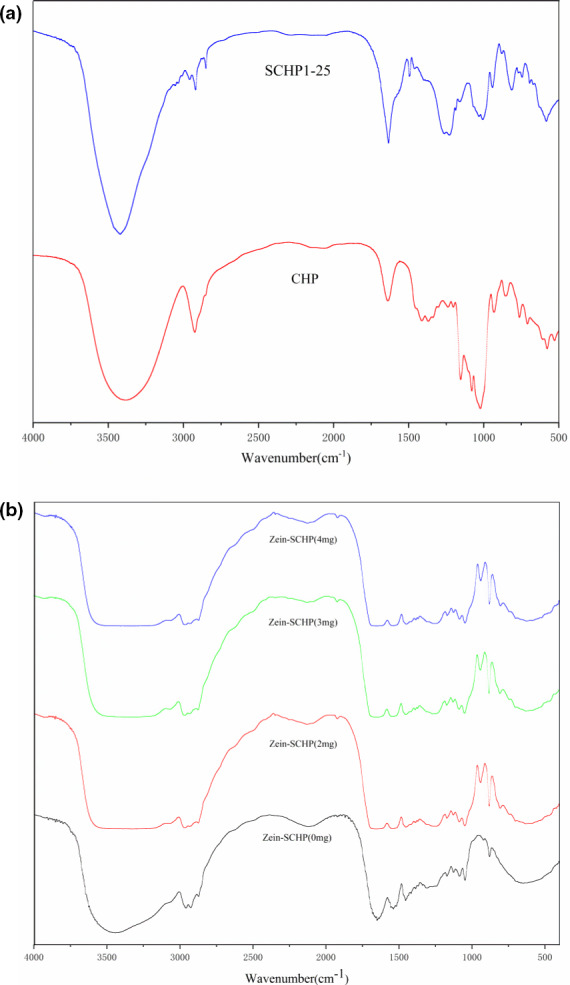
FTIR spectra of (a) Cardamine hupingshanensis polysaccharide and sulfated C. hupingshanensis polysaccharide and (b) Zein‐SCHP films with different concentrations of added SCHP

The FTIR spectra of CHP and SCHP are shown in Figure 2a. It can be seen from the figure that CHP and SCHP have obvious polysaccharide absorption peaks. Among them, the stretching vibration of O‐H caused the absorption peak at 3,419.60 cm^−1^ and the stretching vibration of C‐H caused the absorption peak at 2,918.36 cm^−1^. Compared with CHP, SCHP showed two new absorption peaks at 1,258.61 cm^−1^ and 813.64 cm^−1^, mainly due to the asymmetric S = O stretching vibration in the sulfate group and the symmetric C‐O‐S vibration associated with the C‐O‐SO_3_ group. This also proves that the sulfate group is successfully linked to the CHP. Similar results were observed earlier (Chen et al., 2015). It was found that the absorption peaks due to sulfate groups appearing in 800–850 cm^−1^ can be used to determine the sites where sulfate groups are linked in the long chain of polysaccharides. The sulfate group was determined to be linked at position C‐6 of the long chain of CHP.

The FTIR spectra of Zein‐SCHP and Zein membranes of different concentrations are presented in Figure 2b. First, the FTIR spectra of Zein films were like those of Zein which indicated that the main structure did not change after the formation of Zein film. However, the characteristic peaks were shifted after film formation, which was mainly due to the intermolecular forces during the process of film formation. According to the observations of Surewicz et al. (1993), the absorption peak at 1,700 cm^−1^ is mainly used to study the changes in the secondary structure of proteins and the secondary structures of different peptides and proteins have different vibrational frequencies. It was observed that the characteristic peak of the Zein membrane appeared at 1649.82 cm^−1^ (Figure 2b). The characteristic peak was shifted to the blue region compared to the Zein‐SCHP membrane at different concentrations. The preliminary analysis was mainly due to an increase of intermolecular forces after the addition of SCHP. A new absorption peak was observed at 1,126 cm^−1^ for Zein‐SCHP film (Figure 2b). This indicates that the structure of SCHP did not change during the process of film formation.

### 3.7 *SEM* and AFM of Zein‐SCHP

**FIGURE 3:**
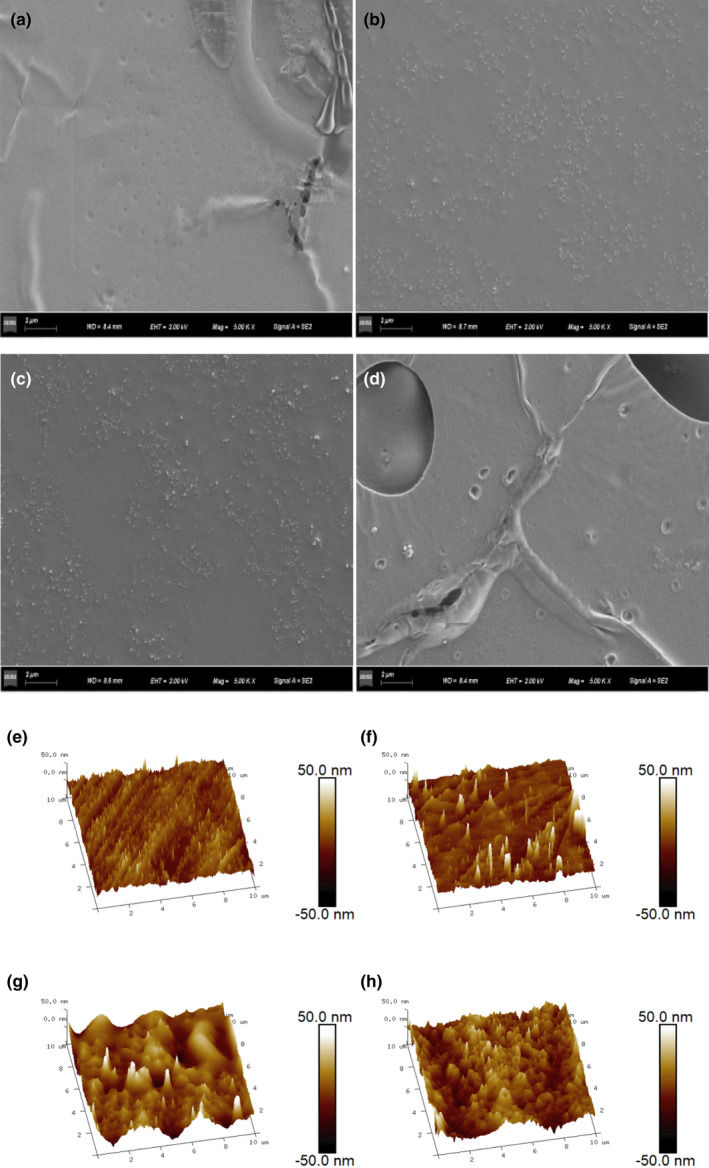
*SEM* topographic images of Zein‐SCHP films. (a) 0 mg/ml SCHP, (b) 1 mg/ml SCHP, (c) 2 mg/ml SCHP, (d) 3 mg/ml SCHP; AFM micrographs of Zein‐SCHP films. Surface: (e) 0 mg/ml SCHP, (f) 1 mg/ml SCHP, (g) 2 mg/ml SCHP, (h) 3 mg/ml SCHP

The *SEM* images of the films containing different SCHP concentrations are presented in Figure 3. Without the addition of SCHP, it was observed that the film was relatively smooth, whereas after the addition of different concentrations of SCHP, several particles appeared on the surface of the film. This could be the reason for the nonsmooth surface of the film. Moreover, as the concentration of added SCHP increased, small pores appeared on the surface of the film at 4 mg/ml. This may be due to the high concentration of supplemented SCHP; thus, creating small pores on the surface of the Zein‐SCHP film. Similar results were observed with the addition of blueberry leaf extract to the polysaccharide film (Han & Song, 2021) and the roughness of the film also increased with the addition of SCHP, as observed from the AFM images of the film (Figure 3e–h). The Image Ra of the film increased from 4.99 nm to 8.93 nm. The Image Rmax of the Zein‐SCHP film with 4 mg/ml SCHP was 202 nm, whereas that of the film without SCHP was 82.1. This also indicates that the roughness of the film surface increases with an increase in the concentration of added SCHP.

### 3.8 Antioxidant properties of the film

**FIGURE 4:**
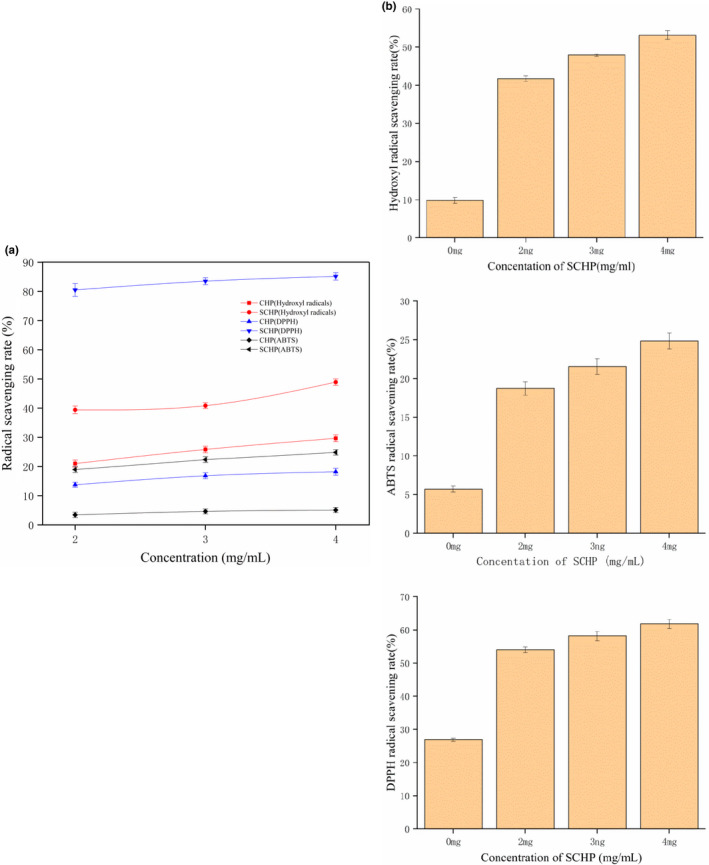
Antioxidant activity of (a) CHP and SCHP and (b) Zein‐SCHP films

It was observed that the ability of SCHP to scavenge hydroxyl radicals, ABTS, and DPPH after modification by sulfation was greatly enhanced with an increasing concentration of SCHP. The scavenging of hydroxyl radicals, ABTS, and DPPH by SCHP at a concentration of 3 mg/ml was enhanced by 14.28%, 13.62%, and 68.33%, respectively (Figure 4a). This also indicates that SCHP can improve the antioxidant capacity of the film. All concentrations of the SCHP added in the Zein‐SCHP membranes significantly increased the antioxidant properties of the membranes. Due to the addition of SCHP (2 mg/ml, 3 mg/ml, and 4 mg/ml), the hydroxyl radical scavenging rate of the membranes was greatly enhanced by 31.97%, 38.12%, and 43.37%, respectively (Figure 4b). The hydroxyl radical scavenging rate in membranes without the addition of SCHP was only 9.78%. The addition of SCHP also enhanced the scavenging rate of ABTS in the membranes. The scavenging rate in membranes without the addition of SCHP was 5.7%, which increased to 18.72%, 21.55%, and 24.84% with the addition of 2 mg/ml, 3 mg/ml, and 4 mg/ml SCHP, respectively. Meanwhile, the DPPH scavenging rate of the membrane after the addition of SCHP addition reached 54.01%, 58.19%, and 61.82%, respectively, which was a two‐ to threefold improvement over the membrane without SCHP addition. The improved antioxidant ability of modified polysaccharides has also been reported by Li et al. (2021). The *R*. *virescens* polysaccharides modified by sulfating showed significantly improved antioxidant properties compared to nonmodified polysaccharides.

## 4 CONCLUSIONS

A novel food packaging composite film was prepared by mixing sulfated CHP with Zein by the liquid–liquid dispersion method, which also had antioxidant properties. The comparison of the microstructure revealed that the surface hydrophobicity of the films supplemented with 4 mg/ml SCHP was enhanced, and the surface roughness was increased. Regarding mechanical properties, Young's modulus of the film increased with an increase in the concentration of supplemented SCHP. In the Zein‐SCHP films, resistance to oxidation increased with an increasing concentration of SCHP. Therefore, Zein‐SCHP is a laminated film that can be used as a food packaging material with good effects on health.

## ACKNOWLEDGEMENTS

None.

## CONFLICT OF INTEREST

The authors declared no potential conflicts of interest.

## Data Availability

The data of this study can be available by contacting the corresponding author.
